# IGAN: An Inception-Based Generative Adversarial Network for Multi-Scale Image Synthesis

**DOI:** 10.3390/jimaging12100518

**Published:** 2026-10-17

**Authors:** Ahmed A. Hashim, Ali Al-Shuwaili, Asraa J. Saeed, Ali Al-Bayaty, Abbas AlZubaidi

**Affiliations:** 1 College of Business Informatics, University of Information Technology and Communications, Baghdad 10015, Iraq; dr.ahmed.hashim@uoitc.edu.iq; 2 College of Engineering, University of Information Technology and Communications, Baghdad 10015, Iraq; ali.najdi@uoitc.edu.iq; 3 Information Institute for Postgraduate Studies, University of Information Technology and Communications, Baghdad 10015, Iraq; phd202110680@iips.edu.iq; 4 Department of Electrical and Computer Engineering, Portland State University, Portland, OR 97201, USA; 5 College of Healthcare Technologies, American University of Iraq–Baghdad, Baghdad 10015, Iraq; abbas.alzubaidi@auib.edu.iq

**Keywords:** Generative Adversarial Networks, image synthesis, inception module, dilated convolution, spectral normalization, dropout, Fréchet Inception Distance, multi-scale representation

## Abstract

Generative Adversarial Networks (GANs) provide a powerful framework for image synthesis, but their practical effectiveness is often limited by the interplay between high visual fidelity and stable adversarial training. Heavily parameterized GANs can improve feature representation; yet, they may also increase the risk of gradient instability or and mode collapse and hence representation design and adversarial optimization must be considered together. This study presents IGAN, a specific Inception-based configuration for unconditional synthesis of 64×64 RGB images. Four-branch modules combine pointwise, local, wider-field, and pooled features in both the generator and discriminator. Bottleneck projections allocate channels before the spatial convolutions; generator modules preserve resolution, whereas discriminator modules reduce it. The documented generator module uses 199,072 convolutional parameters, compared with 590,080 for a corresponding unreduced four-branch block, a 66.26% reduction in parameters count. The model is experimentally evaluated on CUB-200 and selected ImageNet categories. The reported results show Fréchet Inception Distance (FID) values of 13.12 and 15.08 on CUB-200 and ImageNet, respectively, and Inception Scores of 9.27 and 68.25, respectively, compare to certain GAN variations baselines.

## 1. Introduction

Generative Adversarial Networks (GANs) [1] have become a central family of generative models for image synthesis. It typically composed of two neural networks trained through an adversarial objective: a generator *G* maps a random latent vector *z* into a synthetic image, and a discriminator *D* tries to differentiate generated images from real samples. Although this formulation is simple and powerful, GAN training is frequently unstable. The two models optimize coupled objectives, and improvements in discriminator accuracy do not necessarily translate into improved coverage of the data distribution. Training instability and mode collapse are related concerns, but they are not interchangeable: a model can produce a narrow set of plausible images while its scalar losses remain bounded [2,3].

Architectural capacity, receptive-field structure, and optimization are therefore distinct design choices. Increasing width or depth can enrich the function class but also changes memory use and training cost. Conversely, reducing parameters does not by itself preserve image quality or reduce wall-clock time. Convolutional GANs, attention-based GANs, and style-based generators illustrate different choices within this design space [4,5,6]. A useful architectural study must identify the structural change under examination and separate its calculable consequences from benefits that require controlled experiments.

IGAN is motivated by the observation that image synthesis requires simultaneous modeling of local texture, mid-level structure, and broader contextual information. Instead of relying only on sequential convolutional layers, IGAN uses sparse multi-branch modules inspired by the Inception architecture [7,8]. Each module processes feature maps through parallel branches and concatenates the resulting features. Pointwise projections provide channel mixing; bottlenecked 3×3 and 5×5 paths process different spatial neighborhoods; and a pooled path supplies complementary contextual features. The generator’s declared wider branch uses dilation two.

This structure is applied in both networks: the generator and discriminator. In the generator, the inception-inspired modules support multi-scale feature synthesis during progressive upsampling from a 100-dimensional latent vector to a 64×64×3 RGB output image. Here, “progressive upsampling” describes successive spatial expansions within one network, not a progressive-growing training schedule. In the discriminator, corresponding modules reduce spatial resolution while increasing the combined channel width. The design uses established normalization and regularization mechanisms rather than a new adversarial objective.

The research contribution is deliberately bounded. IGAN does not originate Inception modules, multi-scale adversarial learning, dilation, spectral normalization, batch normalization, or dropout. Its design contribution is their particular dual-network arrangement: two resolution-preserving, bottlenecked generator modules and two spatially contracting discriminator modules with different internal channel budgets. We characterize that arrangement through explicit branch dimensions, receptive-field spans, and parameter accounting, and report the original 64×64 synthesis observations on fine-grained bird and selected natural-image data. The analytical comparison isolates the effect of the bottleneck on parameter count, whereas the available experiments do not isolate its effect on synthesis quality. Then, we state the performance gain of the proposal compared to the GAN baselines BigGAN, SNGAN, and SAGAN in Section 4.

Section 2 distinguishes the design from related multi-scale and modern generative models. Section 3 specifies the supported topology, mathematical objective, architectural ambiguities, and evaluation scope. Section 4 analyzes the observations and the additional parameter calculation, followed by limitations and conclusions.

## 2. Related Work

### 2.1. Convolutional GAN Architectures

DCGAN [4] introduced a convolutional design for GANs and demonstrated the effectiveness of replacing fully connected image-generation stages with convolutional layers. Batch normalization and structured convolutional feature learning helped improve early GAN training. LSGAN modified the adversarial loss to reduce gradient saturation and improve training behavior [9]. Subsequent GAN models explored larger architectures, residual blocks, attention mechanisms, and normalization techniques to improve visual quality and training stability.

BigGAN uses class conditioning, residual blocks, orthogonal regularization, self-attention, and spectral normalization [10]. SAGAN introduced self-attention to capture long-range dependencies [5], while SNGAN applied spectral normalization to the discriminator [3]. StyleGAN and StyleGAN2 developed style-based synthesis and improved image quality [6,11]. These models are important methodological references, but their published scores cannot serve as matched measurements for IGAN unless dataset selection, conditioning, resolution, sampling, and evaluation are aligned.

More recent directions change the comparison landscape. Adaptive discriminator augmentation addresses limited-data training through an adaptive augmentation process [12]; StyleGAN-XL addresses synthesis over large, diverse datasets [13]. R3GAN revisits the regularized relativistic objective together with modern residual architectures [14]. These developments show that a contemporary baseline is not simply a larger version of an early convolutional GAN. They also separate training-recipe improvements from feature-module design. IGAN has neither an adaptive augmentation controller nor the R3GAN objective, so improvements demonstrated in those studies must not be attributed to IGAN.

### 2.2. Multi-Scale and Inception-Inspired Modeling

Inception-style architectures use parallel convolutional branches to process information at multiple receptive-field scales [7,8]. In image synthesis, multi-scale processing is important because texture, object parts, and global shapes may emerge at different spatial scales. However, “multi-scale” can refer to different information paths. MSG-GAN passes outputs at multiple generator scales to the discriminator, creating inter-network gradient connections [15]. IGAN instead concatenates multiple receptive-field branches inside individual generator and discriminator stages; it does not specify MSG-GAN’s intermediate-image connections.

A closely related generator design uses a main branch, an auxiliary branch that supplies coarse features, and a gated fusion mechanism [16]. IGAN’s four-path concatenation differs from that main/auxiliary gated interaction, and its discriminator also contains multi-branch stages. This is a difference in topology, not evidence that one fusion strategy is superior. ISA-GAN combines an Inception-based encoder and decoder structure with a self-attention mechanism for image-conditioned facial synthesis [17]. Inception–U-Net adversarial restoration has likewise been studied for snow and rain removal [18]. Those approaches establish that Inception-based adversarial modeling is not new. Their image-conditioned tasks differ from IGAN’s noise-to-image formulation, which has no paired source image, encoder–decoder reconstruction target or specified self-attention mechanism.

Relative to these studies, the present design is characterized by the combination of within-stage four-path fusion in both networks, asymmetric bottleneck widths, generator dilation, and discriminator contraction. The proposal, however, is not the suggestion of a new family of GANs. The design objective is to adapt a shared four-branch topology to resolution-preserving synthesis and spatially contracting discrimination, with separate intermediate channel allocations to support multi-scale representation and control generator-module parameter cost The design objective is to adapt a shared four-branch topology to resolution-preserving synthesis and spatially contracting discrimination, with separate intermediate channel allocations to support multi-scale representation and control generator-module parameter cost that stabilizes training while respecting the mandatory symmetric constraint.

### 2.3. Stabilization and Multi-Learner Paradigms

GAN training can be unstable because the generator and discriminator are optimized simultaneously under a non-stationary objective. Two-time-scale updates, normalization, and regularization address different aspects of this problem [3,19,20]. Spectral normalization controls a matrix norm in the chosen layer parameterization; it is not, on its own, a proof of convergence for an entire network with normalization, branching, and stochastic dropout. Likewise, adding several stabilizers does not demonstrate that their combined effect is beneficial. That question is empirical and motivates the ablation discussed in Section 4.2.

Mutual-guidance and multi-learner methods provide a different form of interaction. Deep Mutual Learning trains a cohort of networks using peer predictions as cooperative supervisory information [21]. IGAN’s generator and discriminator have opposing objectives, and its feature branches are jointly optimized components, not separately supervised learners. Thus, branch diversity should not be conflated with mutual distillation, consensus learning, or a tri-learner system  [22,23]. Cooperative agreement losses could be investigated in a future extension, but none are part of the current objective.

Han et al. [23] analyze adversarial learning, mutual guide learning, and Confucius tri-learning through Vapnik–Chervonenkis bounds, identifying conditions under which additional training samples improve classifier generalization. Confucius tri-learning combines two mutually guiding classifiers with a generator; its analysis concerns interactions among learners, whereas IGAN concerns multi-scale feature allocation within a generator–discriminator pair.

### 2.4. Generative-Model Evaluation and Diffusion-Based Synthesis

Evaluation is sensitive to choices beyond the model architecture. FID was introduced as a feature-distribution comparison [19]; finite-sample bias, resizing, and compression can affect its measured value [24,25]. StudioGAN emphasizes consistent benchmarking across implementations [26]. Precision and recall are separate aspects of sample fidelity and coverage that a single aggregate score may obscure [27]. Recent work has examined limitations of FID’s feature representation and proposed alternative distributional measurements [28]. Reference [29], for instance, explores the impact of reference-set geometry on FID. These findings support explicit reporting of the evaluator and reference set rather than treating a metric name as a complete protocol.

Diffusion models provide a complementary synthesis framework. Denoising diffusion probabilistic models learn a reverse denoising process [30]; latent diffusion moves much of this process into a learned latent representation [31]. Rectified-flow transformers further expand modern image synthesis beyond convolutional adversarial architectures [32]. IGAN generates an image with one generator forward pass after training, whereas a conventional diffusion sampler repeatedly evaluates a denoiser. This procedural difference is not, by itself, a measured latency advantage: image size, model scale, hardware, sampler, and output quality must be matched. Moreover, consistency models support few-step or one-step generation, so iterative sampling is not a universal limitation of contemporary non-adversarial generators [33]. High-resolution generation is yet to be investigated.

## 3. Materials and Methods

### 3.1. Problem Formulation

Let $X\subset R^{H\times W\times C}$ be the image domain, where H=W=64 and C=3 for the RGB images considered in this work. Let $p_{\mathrm{data}}\left(x\right)$ denote the distribution of real images and $p_{z}\left(z\right)$ denote a latent prior, specified in the model as

(1) $$z∼N\left(0,I\right),\;z\in R^{100}.$$

The generator $G_{\theta }$ maps the latent vector to a synthetic image:

(2) $$\hat{x}=G_{\theta }\left(z\right),\;\hat{x}\in R^{64\times 64\times 3}.$$

The discriminator $D_{\phi }$ maps an image to a scalar probability,

(3) $$D_{\phi }\left(x\right)\in \left[0,1\right],$$

where values close to 1 indicate real samples and values close to 0 indicate generated samples. No class label or reference image is supplied as a network input. Training separate models on individual categories is therefore distinct from conditioning a single generator on category labels.

### 3.2. Adversarial Objective

The standard minimax GAN objective is

(4) $$\min_{G}\max_{D}V\left(D,G\right)=E_{x∼p_{\mathrm{data}}}\left[\log D\left(x\right)\right]+E_{z∼p_{z}}\left[\log\left(1-D\left(G\left(z\right)\right)\right)\right].$$

The discriminator loss $L_{D}$ and the non-saturating generator loss $L_{G}$ are

(5) $$\begin{array}{cc} L_{D} & =-E_{x∼p_{\mathrm{data}}}\left[\log D_{\phi }\left(x\right)\right]-E_{z∼p_{z}}\left[\log\left(1-D_{\phi }\left(G_{\theta }\left(z\right)\right)\right)\right], \end{array}$$

(6) $$\begin{array}{cc} L_{G} & =-E_{z∼p_{z}}\left[\log D_{\phi }\left(G_{\theta }\left(z\right)\right)\right]. \end{array}$$

The non-saturating generator loss is commonly used to provide stronger gradients during early training [1]. Both losses in Equations (5) and (6) are minimized with respect to their own parameters. Equation (6) is a surrogate generator objective, not the literal generator part of Equation (4). During a discriminator update, the generated batch is detached from generator optimization; during a generator update the discriminator parameters are held fixed while its input gradient is retained.

### 3.3. Inception-Inspired Feature Module

Let $X\in R^{H\times W\times C}$ denotes the input feature tensor to the IGAN inception module. This module contains four parallel branches. To distinguish topology from activation-placement details, let $T_{k,d,s}^{a\to b}$ denote an affine convolution process with kernel size *k*, dilation *d*, stride *s*, and *a* input and *b* output channels. The branch skeleton is

(7) $$\begin{array}{cc} B_{1} & =T_{1,1,s}^{C\to b_{1}}\left(X\right), \end{array}$$

(8) $$\begin{array}{cc} B_{2} & =T_{3,1,s}^{r_{3}\to b_{2}}\left(T_{1,1,1}^{C\to r_{3}}\left(X\right)\right), \end{array}$$

(9) $$\begin{array}{cc} B_{3} & =T_{5,d_{5},s}^{r_{5}\to b_{3}}\left(T_{1,1,1}^{C\to r_{5}}\left(X\right)\right), \end{array}$$

(10) $$\begin{array}{cc} B_{4} & =T_{1,1,s}^{C\to b_{4}}\left(P(X)\right), \end{array}$$

(11) $$\begin{array}{cc} Y_{\mathrm{cat}} & ={\mathrm{Concat}}_{\mathrm{channels}}\left(B_{1},B_{2},B_{3},B_{4}\right). \end{array}$$

Here, $r_{3}$ and $r_{5}$ are reduction widths, $b_{i}$ are branch output widths, and *P* is the pooling operator. In (7) through (11), all dimensions of height and width are identical: the generator has s=1 while the discriminator needs a factor-two contraction in every completed branch. The reporting order in Equation (11) is pointwise, local, wide, and pooled.

**Figure 1:**
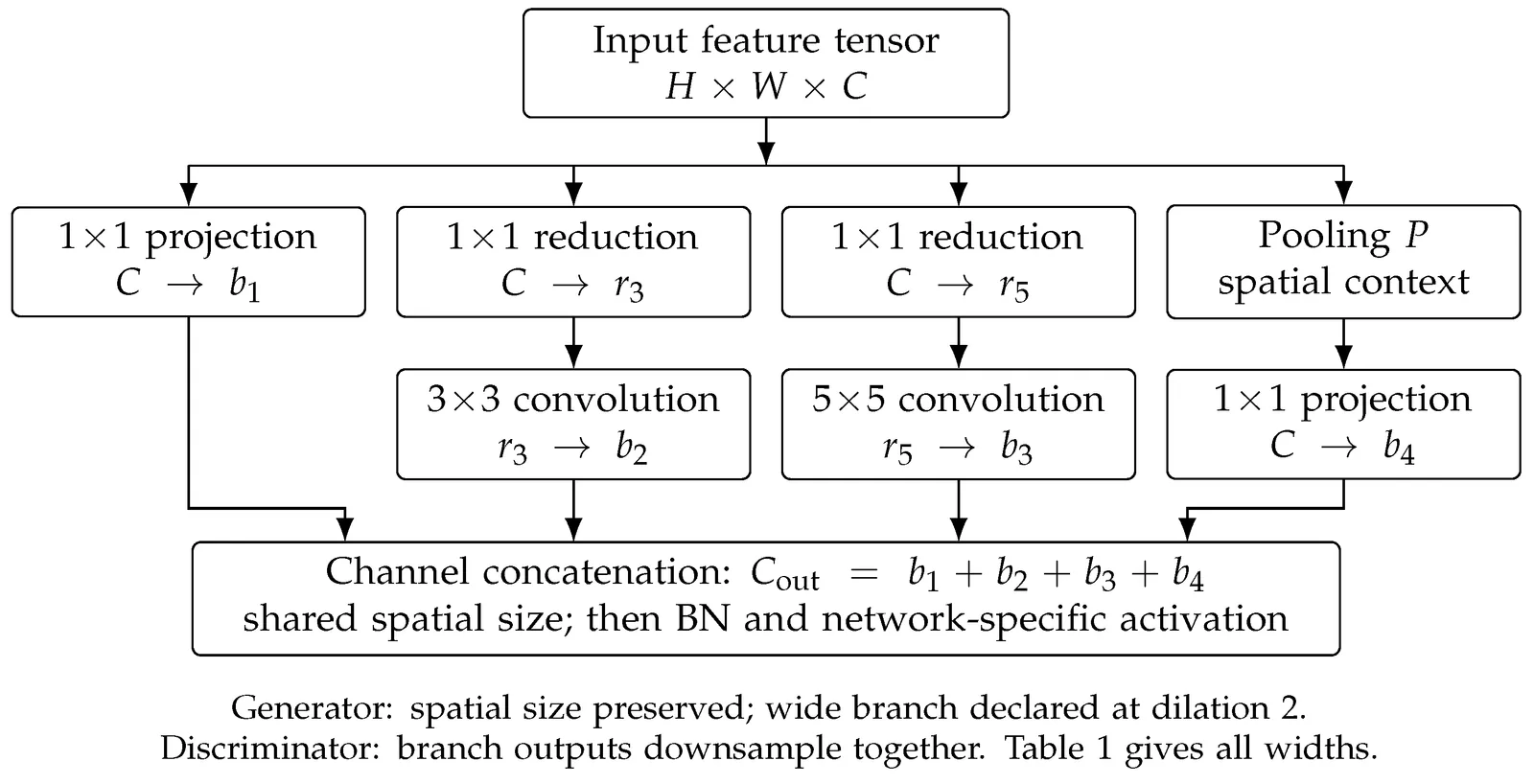
Common four-branch topology depicting the supported affine paths and post-concatenation normalization. Table 1 lists dimensions of the indicated branches.

The dimensions displayed in Figure 1 are detailed in Table 1. Each branch of both generator and discriminator contributes 64 channels, but the local and wider branches reduce to 128 and 32 channels, respectively. D1 uses four 32-channel outputs and D2 four 64-channel outputs. Therefore $C_{\mathrm{out}}=\sum_{i}b_{i}$ is 256 for G1/G2, 128 for D1, and 256 for D2.

#### Branch Dimensions and Spatial Compatibility

For an input spatial extent *n*, a convolution with total padding $p_{\mathrm{tot}}$ produces

(12) $$n_{\mathrm{out}}=\left\lfloor \frac{n+p_{\mathrm{tot}}-d\left(k-1\right)-1}{s}\right\rfloor +1.$$

For stride one, size preservation requires $p_{\mathrm{tot}}=d\left(k-1\right)$; a 5×5 kernel at dilation two therefore needs eight total padding positions per dimension. The effective kernel span is $k_{\mathrm{eff}}=1+\left(k-1\right)d$, giving a nine-position span for this branch without changing its 25 kernel coefficients per input/output channel pair. This sparse span is not a dense 9×9 convolution and does not imply equal expressiveness. For the downsampling modules, the spatial sizes in Table 2 require compatible padding and a common factor-two output reduction.

### 3.4. Spectral Normalization and Regularization

Spectral normalization [3] normalizes a weight matrix W by its largest singular value:

(13) $$\bar{W}=\frac{W}{\sigma_{\max}\left(W\right)}.$$

This controls the chosen matrix representation. It establishes that the complete discriminator is globally 1-Lipschitz. SN is specified at the two non-RGB outer generator convolutions.

Batch normalization [20] transforms an activation as

(14) $$\mathrm{BN}\left(x\right)=\gamma \frac{x-\mu_{B}}{\sqrt{\sigma_{B}^{2}+\epsilon }}+\beta .$$

**Figure 2:**
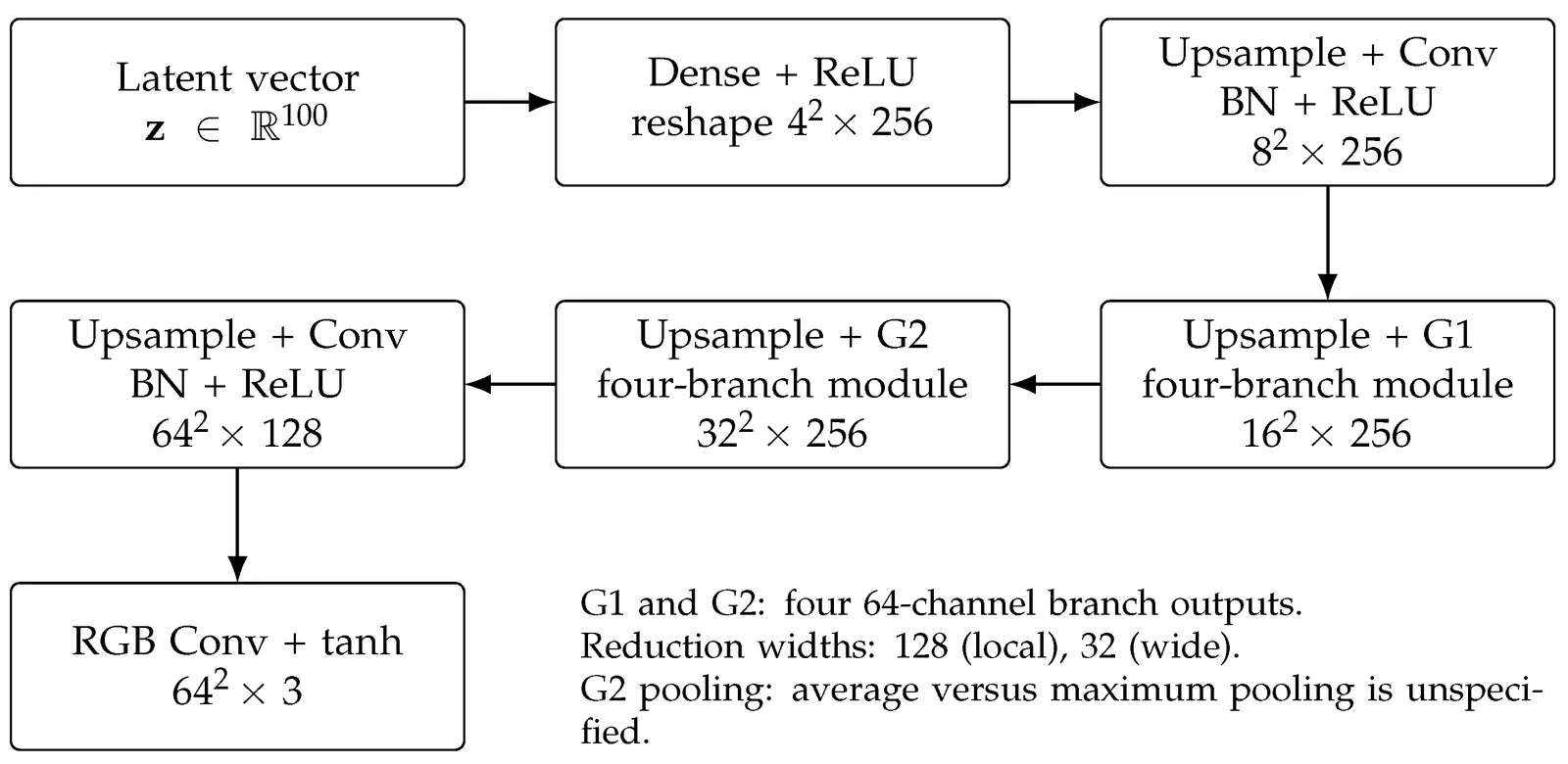
Concise generator schematic. G1 and G2 use the branch layout in Figure 1; exhaustive widths and counts appear in Table 1 and Table 2.

**Figure 3:**
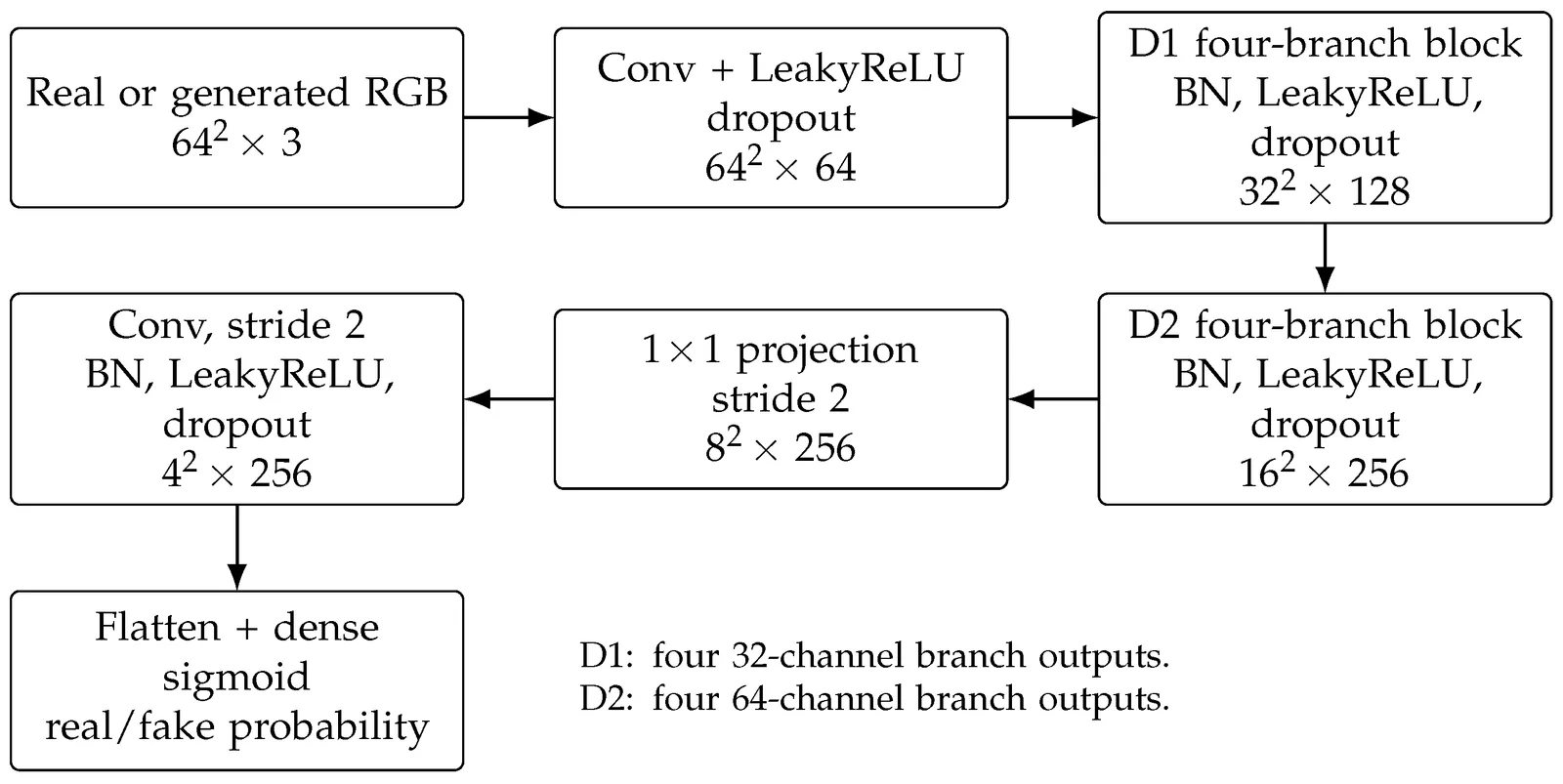
Concise discriminator schematic. Branch concatenation occurs only after spatial alignment. Normalization and dropout placement follow the displayed graph; numerical settings and full SN coverage are unspecified.

The generator graphs place BN followed by ReLU after each module concatenation. The discriminator graphs place BN, LeakyReLU, and dropout in that order after each module, with further regularization after the final spatial convolution. The input discriminator convolution is followed by LeakyReLU and dropout without a displayed BN layer. BN’s moving-statistic convention, LeakyReLU slope, dropout rate and scaling convention, and evaluation-mode behavior are specified in Figure 2 and Figure 3.

### 3.5. Generator Architecture

The generator converts a vector containing 100 latent values into a 64×64×3 RGB image. A fully connected layer maps the input to 4096 features, which are reshaped to 4×4×256. The graph includes ReLU at this initial dense stage. Successive twofold upsampling stages produce 8×8, 16×16, 32×32, and 64×64 features. A 3×3 convolution precedes the two Inception modules; a further 3×3 convolution reduces the final feature width to 128. The output convolution maps those features to three channels with tanh activation.

### 3.6. Discriminator Architecture

The discriminator side receives a real or generated 64×64×3 image and outputs a scalar probability. The initial 3×3 convolution produces 64 channels without reducing spatial resolution. D1 then produces 32×32×128 features, and D2 produces 16×16×256 features. A stride-two 1×1 projection and a stride-two 3×3 convolution reduce the features to 8×8×256 and 4×4×256, respectively. Flattening yields 4096 inputs to the final sigmoid unit. Unlike generator upsampling, this contraction progressively aggregates evidence for the real/fake decision. Its detailed branch widths are asymmetric: D1’s local and wide reductions both have 32 channels, while D2 uses 128 and 64.

### 3.7. Parameter Accounting and Analytical Bottleneck Comparison

A biased k×k convolution from *a* input to *b* output channels has $k^{2}ab+b$ trainable coefficients. BN with *b* output channels contributes 2b trainable affine coefficients and 2b running-statistic values under the convention used here. For example, the expression “590,080 + 1024” consists of 590,080 convolutional coefficients and 1024 BN values. From which, 512 are trainable, and 512 are running state. Its combined total is 591,104, not 591,104 trainable parameters. Table 2 separates these quantities throughout.

For a four-branch block with widths defined above, its convolutional count is

(15) $$\begin{array}{cc} N_{\mathrm{conv}}= & \left(Cb_{1}+b_{1}\right)+\left(Cr_{3}+r_{3}\right)+\left(9r_{3}b_{2}+b_{2}\right)\\ \; & +\left(Cr_{5}+r_{5}\right)+\left(25r_{5}b_{3}+b_{3}\right)+\left(Cb_{4}+b_{4}\right). \end{array}$$

For G1 or G2, the four path totals are 16,448, 106,688, 59,488, and 16,448, summing to 199,072. Including its 512 BN affine coefficients gives 199,584 trainable parameters. The corresponding convolutional counts for D1 and D2 are 43,200 and 217,536. Summing the network stages yields 1,702,211 generator and 923,777 discriminator trainable parameters, under the stated bias and BN conventions. BN running state is counted separately; framework counters and spectral-normalization are independently recomputed analytical quantities.

A local structural comparison isolates the bottleneck’s effect. Keep the four 64-channel generator outputs but remove the reduction projections before the 3×3 and 5×5 convolutions. Directly processing all 256 input channels then requires

(16) $$N_{\mathrm{unreduced}}=\left(1+9+25+1\right)\;25,664+4\left(64\right)=590,080.$$

The reduction is 1−199,072/590,080=66.26%. Both variants have the same concatenated width and would have the same post-concatenation BN count, but their function classes differ. In particular, the reduced paths constrain intermediate channel mixing. This arithmetic establishes a parameter tradeoff.

### 3.8. Training Procedure

Algorithm 1 presents the alternating optimization steps. It is a statement of the objective-level procedure. The symbols *T*, *m*, and $n_{D}$ identify the total update cycles, batch size, and discriminator-to-generator update ratio.
**Algorithm 1** IGAN alternating non-saturating optimization (objective-level specification)**Require:** Training-image distribution, initialized $G_{\theta },D_{\phi }$, batch size *m*, update count *T*, discriminator ratio $n_{D}$, configured optimizers  1:**for** t=1,…,T **do**  2:   **for** $j=1,\ldots ,n_{D}$ **do**  3:      Sample real images ${\left\{x_{i}\right\}}_{i=1}^{m}$ and latent vectors ${\left\{z_{i}\right\}}_{i=1}^{m}$  4:      Compute ${\hat{x}}_{i}=G_{\theta }\left(z_{i}\right)$ and detach from generator optimization  5:      Estimate $L_{D}$ by the minibatch form of Equation (5)  6:      Update ϕ to minimize $L_{D}$; leave θ unchanged  7:   **end for**  8:   Sample a new latent minibatch and evaluate $D_{\phi }\left(G_{\theta }\left(z_{i}\right)\right)$  9:   Update θ to minimize Equation (6); do not update ϕ10:   Apply the configured normalization-state and checkpoint policies11:**end for**

### 3.9. Datasets and Experimental Protocol

The IGAN model is experimentally evaluated on the CUB-200 dataset and a selected subset of ImageNet, with individual generated-image examples for “Tiger, Saint Bernard, Geyser, Redshank, Bee, Ant, Valley, and Goldfish”. The generated images are reported at 64×64 RGB. The ImageNet description reports 20 pooled categories containing 10,336 images and additional category-specific training for the eight named categories. The remaining 12 categories, synset identifiers, and per-category counts are not identified. Therefore, the pooled selection cannot be treated as either all of ImageNet or a reproducibly defined benchmark subset.

The cited bird dataset is CUB-200-2011 [34], whose official release contains 200 categories. We use the shortened designation “CUB-200”. ImageNet is cited to its original dataset description [35].

### 3.10. Evaluation Metrics and Interpretation

#### 3.10.1. Fréchet Inception Distance

The Fréchet Inception Distance (FID) [19] compares the distribution of features extracted from real and generated images. We denote by $\left(\mu_{r},\Sigma_{r}\right)$ and $\left(\mu_{g},\Sigma_{g}\right)$ the mean and covariance of both real and generated image features, respectively. A symmetric positive-semidefinite form is

(17) $$\mathrm{FID}={∥\mu_{r}-\mu_{g}∥}_{2}^{2}+\mathrm{Tr}\left(\Sigma_{r}+\Sigma_{g}-2{\left(\Sigma_{r}^{1/2}\Sigma_{g}\Sigma_{r}^{1/2}\right)}^{1/2}\right).$$

Lower FID indicates closer agreement under the chosen feature-distribution approximation, not necessarily better coverage or perceptual quality in every setting. The symmetric form avoids interpreting a generally nonsymmetric covariance product as a symmetric matrix. Sample size and low-level image processing must still be controlled [24,25].

#### 3.10.2. Inception Score

The Inception Score (IS) [2,36] is

(18) $$\mathrm{IS}=\exp\left(E_{x∼p_{g}}\left[D_{\mathrm{KL}}\left(p(y|x)\;∥\;p(y)\right)\right]\right),$$

with p(y∣x) being the classifier distribution predicted for a generated image and p(y) is its marginal. IS does not compare generated samples with the real reference images. Its information-theoretic form is logIS=H(Y)−H(Y∣X); hence, for a classifier with *K* outputs, 1≤IS≤K.

## 4. Results and Discussion

Our introduced IGAN is trained on the two mentioned datasets: (i) CUB-200 [34]; (ii) ImageNet [35] with a total of twenty distinct categories, from which IGAN is trained on eight categories of “Tiger, Saint Bernard, Geyser, Redshank, Bee, Ant, Valley, and Goldfish”; the remaining 12 categories are not identified. All considered images were resized to 64×64 pixels. No cropping or data augmentation was applied. For CUB-200, pixel values were scaled to [0, 1] by dividing by 255. The CUB-200 dataset was splitted as 80% training, 10% validation, and 10% testing. Throughout the results, 10,365 real and 10,365 generated ImageNet images, while 10,000 real and 10,000 generated CUB-200 images were used.

**Figure 4:**
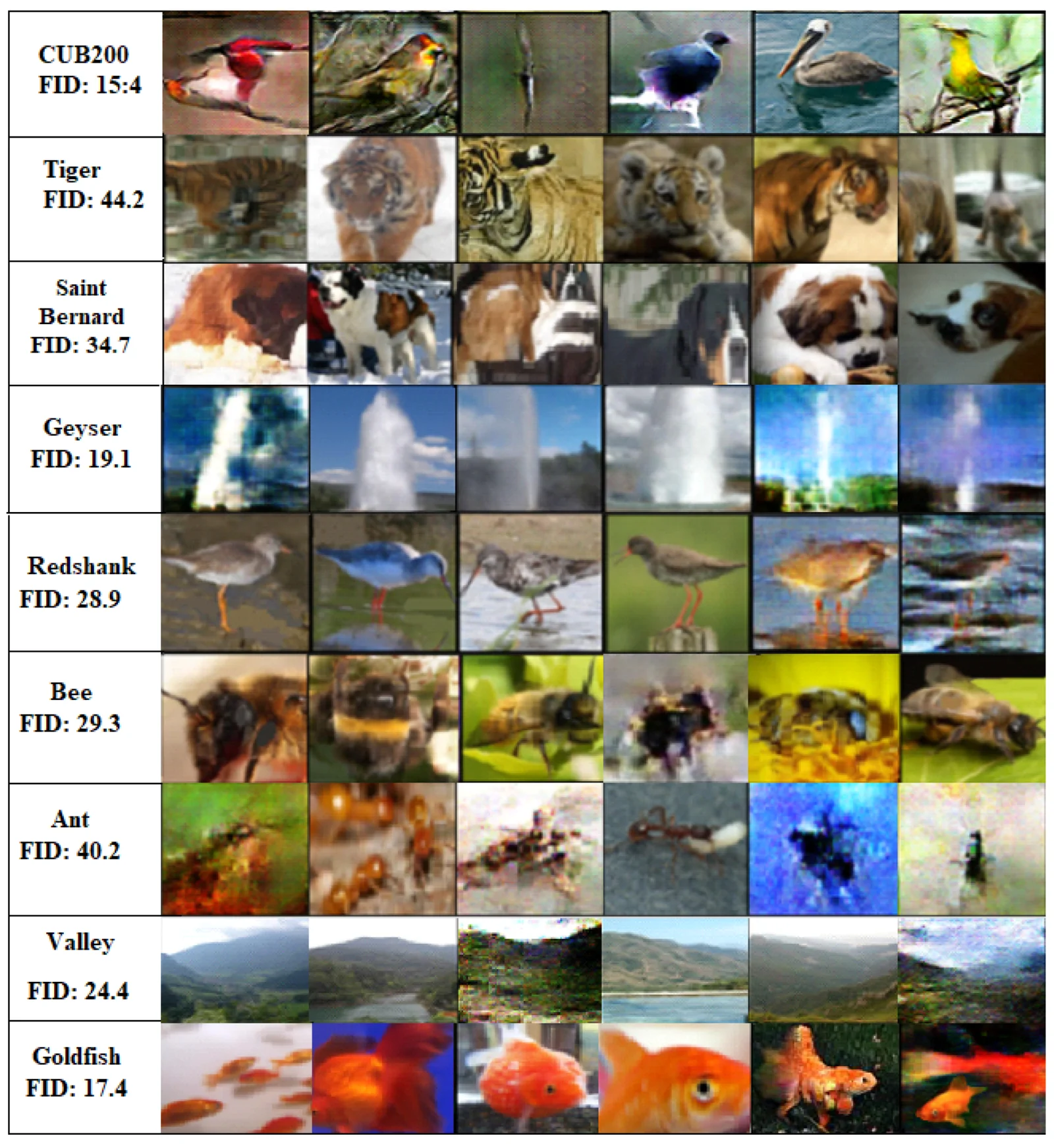
Sample image generation by the IGAN along with FID values.

The obtained generated images are highly realistic, as shown in Figure 4. This improvment is mainly attributed to the stable image synthesis, that requires multi-scale feature extraction and stable adversarial optimization, currently established in the IGAN. Our suggested architecture addresses the feature-extraction component by introducing inception-style parallel branches. The 1×1 branch enables channel mixing and controls computational cost; the 3×3 branch provides local texture modeling; the 5×5 dilated branch increases the effective receptive field; and the average-pooling branch contributes contextual aggregation. The discriminator benefits from the same multi-scale principle because it can evaluate generated images at multiple feature scales.

**Figure 5:**
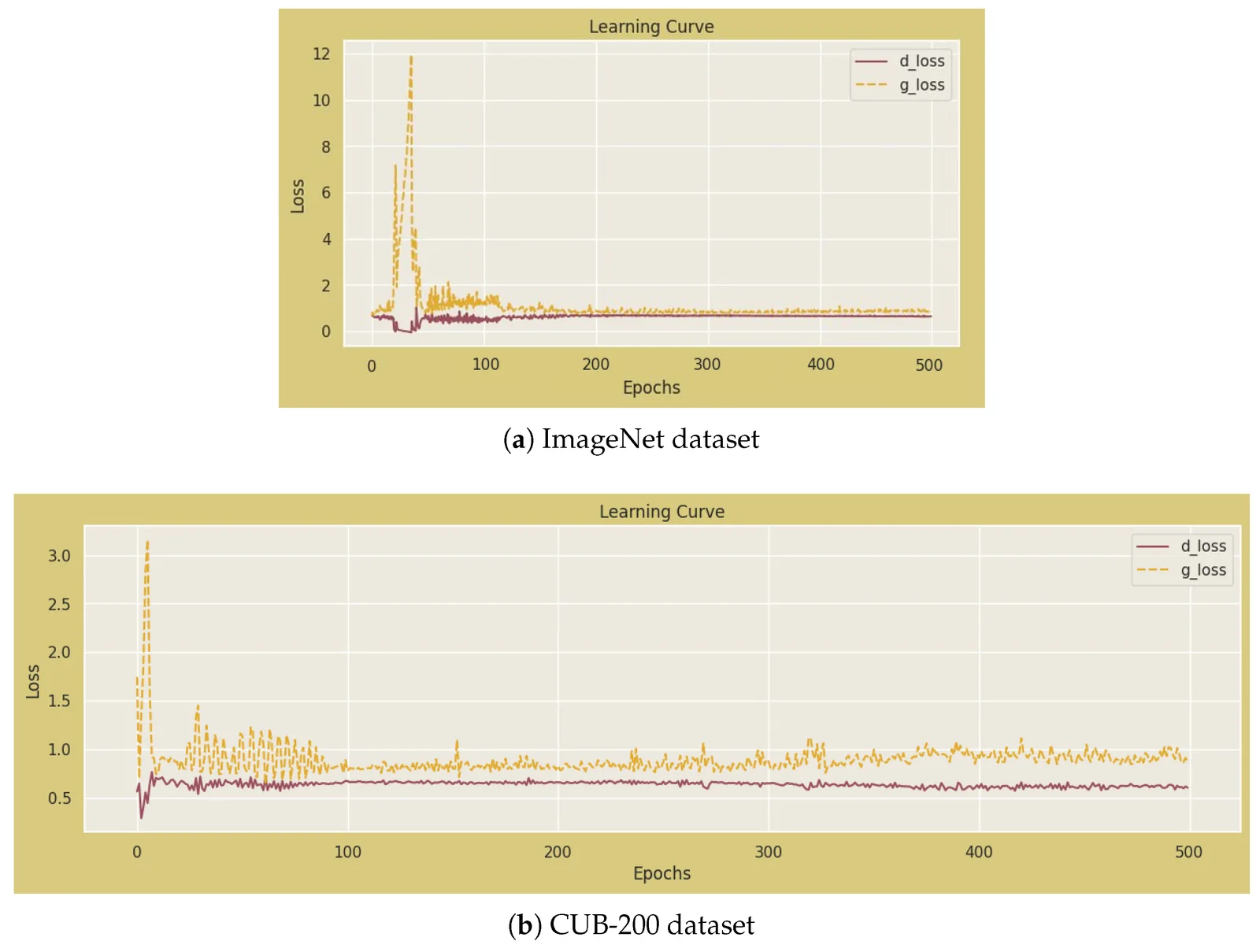
Loss function curves over the 500 Epochs using IGAN for the discriminator ($L_{D}$ as d_loss) and generator ($L_{G}$ as g_loss) on the CUB-200 and twenty classes aggregated of ImageNet datasets.

Figure 5 depicts the loss functions of the generator $G_{\theta }$ and discriminator $D_{\phi }$ networks of the IGAN Algorithm 1 over 500 epochs using: (i) ImageNet consisting of 10,336 images; and (ii) the CUB-200 dataset. Both loss functions $(L_{D}$ and $L_{G}$) reflect considerable degree fluctuations at the beginning of training. The discriminator’s loss $L_{D}$ stabilizes rapidly, indicating its increasing proficiency in discriminating genuine from fake samples. On the contrary, the loss function behavior of the generator $L_{G}$ has a more pronounced level of fluctuations, which then stabilizes, signifying the improved generator capability for producing more authentic data that may successfully mislead the discriminator. Combining the previous two observations, it is evident that the adversarial training dynamics, in both networks, consistently adjust and enhance their performance in reaction to one another. Note that Figure 5b reflects a more stable and effective way to train the generator due to the existence of 10,000 images in only one class. To strengthen the generated image quality, training is performed over a total of 500 iterations.

Our suggested IGAN is first compared with well-known variants of GAN models, namely BigGAN, SNGAN, and SAGAN. This is because these are well-established algorithms in the field of image generation, which aligns with the typical data type used in this IGAN algorithm, allowing for a fair comparison [3,5,10] as reported in Table 3. In Table 3, all FID and IS values demonstrate that the IGAN attain the best values in both metrics.

In Table 3, these results indicate that IGAN achieves the lowest FID among the listed models on both datasets and the highest IS on both datasets. On CUB-200, IGAN improves FID from 18.30 for BigGAN to 13.12. On ImageNet, IGAN improves FID from 22.77 for BigGAN to 15.08. These results correspond to the approximate 28–33% FID improvement relative to the strongest baseline listed for each dataset. In addition, the table reports the available literature-based generator parameter counts and FLOPs. Generator complexity is presented because image synthesis requires only the generator. Differences in output resolution, conditioning, architecture variant, and operation-counting scope limit direct comparison with IGAN. Unavailable numerical values are marked NR rather than estimated from different implementations. Consequently, these entries provide architectural context and do not establish relative inference time or a standardized FLOPs reduction.

Table 4 places the IGAN scores alongside reported results for additional GAN models. Direct FID and IS comparisons require aligned data distributions and evaluation settings; the heterogeneous entries in this table therefore provide context. The CUB-200 ablations in Table 5 and Table 6 provide within-study evidence for the component configuration, while Section 3.7 quantifies the parameter saving. The table also includes available generator complexity, in terms of parameter counts and FLOPs totals. The displayed comparision provide context rather than a standardized computational-efficiency ranking.

### 4.1. Expected Effects on Mode Collapse

Under the bias and BN conventions of Section 3.7, the generator and discriminator contain 1,702,211 and 923,777 trainable parameters, respectively (Table 2). At the stated layer resolutions, one forward evaluation requires 1,514,553,344 dense/convolution multiply–accumulates (MACs) for the generator and 151,785,472 for the discriminator. Counting two floating-point operations per MAC gives approximately 3.0291 and 0.3036 GFLOPs, respectively. These analytical counts exclude bias additions, normalization, activations, pooling, upsampling, spectral-normalization updates, and memory transfers. The comparison of branch configurations suggests that G1 and G2 achieve a more economical use of convolutional parameters while still producing four groups of output features. This reduction is mainly due to the restricted intermediate channel widths. It should not be attributed to dilation, since a dilated 5×5 convolution contains the same number of learnable coefficients as a conventional 5×5 kernel; dilation only changes how those coefficients are distributed over the input. Accordingly, the parameter saving applies to this part of the architecture and should not be interpreted as evidence of an overall reduction in computational cost. Actual runtime can also be affected by normalization, activations, upsampling operations, implementation details, and memory access.

The multi-branch design may also affect training behavior in several ways. In the discriminator, features obtained from different spatial scales can provide complementary information about local detail and broader structure. In the generator, multiple paths allow information from different receptive fields to be combined when constructing image content. Normalization and dropout may further influence optimization by controlling update sensitivity and reducing dependence on a small subset of features. However, these effects should be interpreted cautiously. Different branches can learn overlapping information, dilation can leave gaps in spatial sampling, pooling may remove fine details, and excessive regularization can weaken informative discriminator feedback. None of these mechanisms directly guarantees that all modes of the data distribution will be represented.

The present results therefore support the view that the proposed architecture is designed to improve feature representation and training stability, but they do not demonstrate that any individual component, or the full combination of components, eliminates mode collapse. Establishing such a relationship would require controlled ablation studies together with metrics that evaluate both image quality and distributional coverage. FID and IS alone should also be interpreted with care, since improved aggregate scores do not necessarily imply that all modes of the training distribution are adequately represented.

### 4.2. Ablation Study

For the ablation study, separate IGAN variants will be trained by removing only one component of the Inception module at a time, i.e., the 1 × 1 convolution, 3 × 3 convolution, dilated 5 × 5 convolution, or average-pooling layer as reported in Table 4. To ensure a controlled comparison, the removed component will be replaced with an alternative layer/branch so that the output dimensionality and the number of trainable parameters are preserved as closely as possible. All other architectural and training settings will remain unchanged as shown in Table 5. Importantly, this layer removal-and-replacement procedure will be applied consistently to both the generator and discriminator networks. Thus, each ablation variant will maintain the same input/output dimensions and overall network structure, allowing the observed performance differences to be attributed specifically to the contribution of the corresponding Inception component.

### 4.3. Limitations

The study is limited to 64×64 RGB output; it does not establish performance at resolutions needed for fine-detail or domain-specific applications. The bird sample and selected ImageNet categories provide limited evidence of generalization. Finally, analytical parameter counts do not establish training cost, generation throughput, or deployment memory. Higher resolution, attention, conditional generation, and scientific-image applications remain untested extensions, not current capabilities. It is worth to mention that asymmetric Inception configurations in both generator and discriminator caused tensor-shape incompatibilities that halted training, preventing their evaluation in the current implementation.

## 5. Conclusions

This paper presents IGAN, an inception-inspired GAN architecture for multi-scale image synthesis at 64×64 resolution. The model combines pointwise convolution, local convolution, wider-field processing, pooling, and feature concatenation within generator and discriminator modules. Its specific contribution is the dual-network arrangement and asymmetric bottleneck allocation of established components. The branch dimensions support a interesting analytical insight: a generator Inception block contains 199,072 convolutional parameters compared with 590,080 in its unreduced four-branch counterpart. Numerically, the IGAN model achieves the FID of 13.12 on the CUB-200 dataset and 15.08 on the ImageNet dataset, representing a 28–33% FID improvement as compared to the considered GAN variants baselines, with the IS of 9.27 and 68.25, respectively.

Future work can extend IGAN in several directions. First, the model can be evaluated at higher resolutions using progressive training or modern upsampling blocks. Second, attention mechanisms can be added to improve long-range dependency modeling. Third, conditional generation can be introduced to improve class-specific control. Finally, the architecture can be tested on domain-specific datasets, including medical, remote-sensing, industrial, or scientific imaging data. These extensions should follow a reproducible baseline evaluation rather than substitute for it.

## Figures and Tables

**Table 1:** Branch allocations. “Reduction” is the output width of the preceding 1×1 convolution. The pooling path preserves the input channel count before its output projection.

Module	*C*	$b_{1}$	$r_{3}$	$b_{2}$	$r_{5}$	$b_{3}$	$b_{4}$	$C_{\mathrm{out}}$
G1	256	64	128	64	32	64	64	256
G2	256	64	128	64	32	64	64	256
D1	64	32	32	32	32	32	32	128
D2	128	64	128	64	64	64	64	256

**Table 2:** Trainable, BN state, and combined parameter counts for both generator and discriminator stages.

Network/Stage	Output Shape	Trainable	BN State	Combined
G: dense, ReLU, reshape	4×4×256	413,696	0	413,696
G: upsample, 3×3 conv, BN, ReLU	8×8×256	590,592	512	591,104
G: upsample, G1	16×16×256	199,584	512	200,096
G: upsample, G2	32×32×256	199,584	512	200,096
G: upsample, 3×3 conv, BN, ReLU	64×64×128	295,296	256	295,552
G: 3×3 RGB conv, tanh	64×64×3	3459	0	3459
Generator total		1,702,211	1792	1,704,003
D: 3×3 conv, LeakyReLU, dropout	64×64×64	1792	0	1792
D: D1, BN, LeakyReLU, dropout	32×32×128	43,456	256	43,712
D: D2, BN, LeakyReLU, dropout	16×16×256	218,048	512	218,560
D: 1×1 conv, stride 2	8×8×256	65,792	0	65,792
D: 3×3 conv, stride 2, BN	4×4×256	590,592	512	591,104
D: flatten, dense, sigmoid	1	4097	0	4097
Discriminator total		923,777	1280	925,057

**Table 3:** FID and IS performance of IGAN compared to GAN-variant baselines on CUB-200 and ImageNet datasets. For FID, lower scores are better while for IS, higher scores are generally better. Available generator parameter counts and FLOPs are included for the published configurations specified below.

Model	CUB-200 Dataset		ImageNet Dataset		Generator Complexity	
	IS	FID	IS	FID	Parameters (M)	GFLOPs
BigGAN ^a^	4.98	18.30	38.05	22.77	70.4	NR
SNGAN ^b^	5.41	47.75	32.25	26.79	42.0	9.10
SAGAN ^b^	5.48	54.29	29.85	34.73	42.0	9.18
IGAN ^c^	9.27	13.12	68.25	15.08	1.7022	3.0291

^a^ BigGAN, 128×128. ^b^ SNGAN-projection/SAGAN, 128×128. ^c^ IGAN, 64×64: two FLOPs per dense/convolution MAC. M: million; NR: not reported.

**Table 4:** Reported FID and IS values for GAN models on the listed datasets; direct comparison requires matched evaluation settings. Available generator parameter counts and FLOPs are also reported.

Model	FID	IS	Dataset	G Parameters (M)	G GFLOPs
R3GAN	2.09	-	ImageNet (64×64)	103.57	NR
Hindi T2I GAN	37.17	4.65	CUB	NR	NR
DCGAN Variants	52	4.1	CUB-200-2011	NR	NR
IGAN	13.12	9.27	CUB-200	1.7022	3.0291
IGAN	15.08	68.25	ImageNet	1.7022	3.0291

G: generator; M: million; NR: not reported in the identified source.

**Table 5:** Ablation study on the CUB-200 dataset with stabilization mechanisms, where ✔ means applicable and × means non-applicable.

Configuration	Inception Layer 1	Inception Layer 2	SN	BN	Dropout	FID
Full IGAN	✔	✔	✔	✔	✔	13.12
w/o Inception layer 2	✔	×	✔	✔	✔	unstable
w/o Inception layer 1	×	✔	✔	✔	✔	unstable
w/o BN	✔	✔	✔	×	✔	16.03
w/o SN	✔	✔	×	✔	✔	19.11
w/o Dropout	✔	✔	✔	✔	×	14.2

**Table 6:** Ablation study on CUB-200 dataset with IGAN variants, where × means non-applicable.

Configuration	FID	IS	Add an Alternative Layer
Full IGAN	13.12	9.27	×
IGAN w/o 1×1	15.80	8.91	Add 5×5
IGAN w/o 3×3	14.70	9.02	Add 1×1
IGAN w/o 5×5	18.40	8.51	Add 1×1
IGAN w/o Average-pooling	16.20	8.73	Add 1×1

## Data Availability

The original contributions presented in this study are included in the article. Further inquiries can be directed to the corresponding author.
